# Noncanonical GA and GG 5′ Intron Donor Splice Sites Are Common in the Copepod *Eurytemora affinis*

**DOI:** 10.1534/g3.117.300189

**Published:** 2017-10-27

**Authors:** Hugh M. Robertson

**Affiliations:** Department of Entomology, University of Illinois at Urbana-Champaign, Urbana, Illinois 61801

**Keywords:** noncanonical intron donor splice sites, copepod genome, *Eurytemora*, intron evolution, GA donors

## Abstract

The noncanonical 5′ intron donor splice sites GA and GG are exceedingly rare in described eukaryotic genomes; however, they are present in ∼12% of introns in the genome of the copepod *Eurytemora affinis*. Failure to recognize the high frequency of these donor sites compromised the modeling of genes in this newly sequenced genome, including 10 conserved ionotropic glutamate receptor (GluR) family genes curated herein. These introns appear to have been acquired recently, along with many additional idiosyncratic introns. Their high frequency implies the evolution of modified intron donor splice site recognition in this copepod.

The canonical sequence for 5′ intron donor splice sites in eukaryotes has an obligate G to start the intron followed by U, or occasionally C, as part of a larger consensus splice site sequence, with DNA sequence AG/GTAAGT (Mount 1982). For example, among 222,263 introns in the human genome, Parada *et al.* (2014) found only 184 noncanonical splice sites, of which only 14 were GA and 32 were GG donors (0.006 and 0.014%, respectively). Most of these are sites of alternative splicing, as are the most intensively studied GA donors, belonging to the vertebrate fibroblast growth factor receptor 1–3 genes (Brackenridge *et al.* 2003).

Eyun *et al.* (2017) examined the evolution of arthropod chemosensory genes, focusing on crustaceans and particularly the genome of the copepod *Eurytemora affinis*, one of the first copepod genomes to be sequenced. Among others, the authors reported nine Ionotropic Receptor (IR) genes and five members of the ionotropic GluR family, from which the IRs evolved (Benton *et al.* 2009); however, the amino acid sequences they provided for these proteins are almost all truncated at one or both ends. I have completed the gene models for five conserved members of the IR family and the five GluR family members, and found that they contain an unusually high frequency of noncanonical GA and GG 5′ intron donor splice sites. A sample of 26 other large genes indicates that this unusual phenomenon is likely to be genome-wide.

## Materials and Methods

Gene models were built manually in the text editor TEXTWRANGLER using genomic sequences from the assembly published in Eyun *et al.* (2017) and presented in the i5k Workspace@NAL genome browser (Poelchau *et al.* 2015; https://i5k.nal.usda.gov/). RNAseq reads spanning each intron were either obtained from the i5k genome browser if there was RNAseq mapped across an intron, or from the Short Read Archive (SRA) at the National Center for Biotechnology Information (NCBI) using BLASTN searches with flanking exon sequences as queries and default parameters. Exons missing in assembly gaps were recreated using raw genome reads from the SRA, using the RNAseq reads obtained above as queries. Models were compared with the protein sequences reported by Eyun *et al.* (2017); however, these appear to have been derived from a transcriptome rather than from the genome itself, because they contain descriptors that indicate they were derived from transcripts from CUFFLINKS or another transcriptome assembly (*e.g.*, EaffNMDAR2-1 comp31752_c0_seq1 CUFF.1933.3). They also commonly span both GA and GG donor introns as well as misassembled exons and exons missing from the assembly (see, for example, description of IR25a in Supplemental Material, File S1). Sequence logos for exon–intron junctions were built at http://weblogo.berkeley.edu/logo.cgi.

### Data availability

The sequences of the 10 focus proteins are presented in File S1, as are the nucleotide sequences surrounding the 36 noncanonical intron 5′ splice sites.

## Results and Discussion

**Figure 1:**
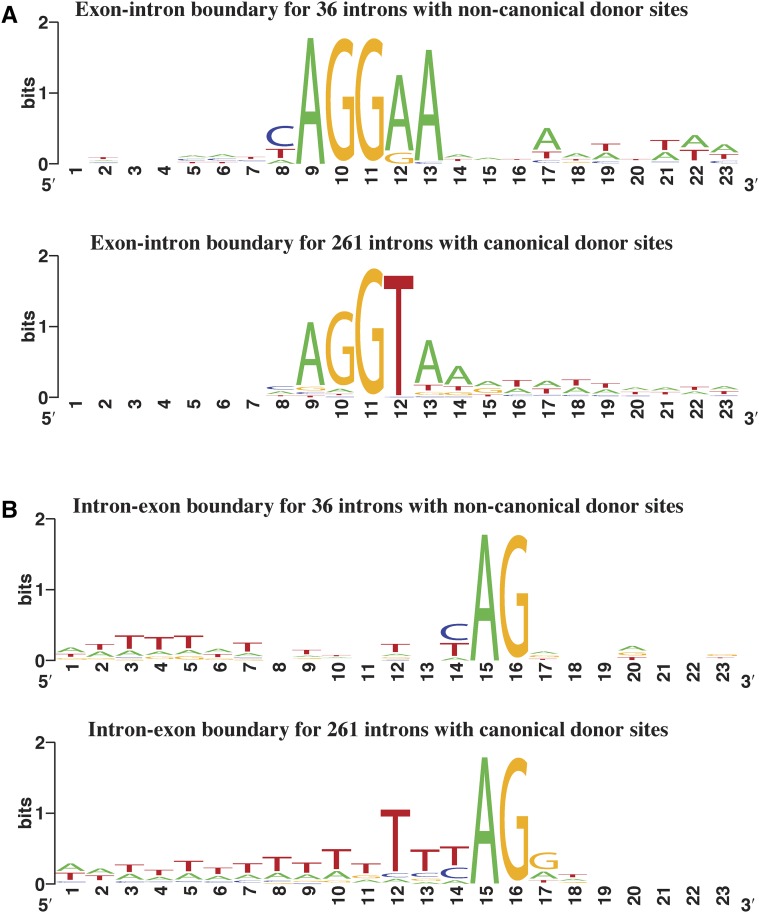
Sequence logos showing information content for the 36 noncanonical GA and GG 5′ intron donor splice sites and the 3′ acceptor sites for these introns, compared with sites for 261 introns with canonical donors in 10 conserved large ionotropic glutamate receptor family genes in the copepod *E. affinis*. (A) Ten bases of exon and 13 bases of intron sequence are shown for the donors. (B) Sixteen bases of intron and seven bases of exon sequence are shown for the acceptors. Sequence logos for frequencies of nucleotides are shown in Figure S1.

The five conserved members of the IR family (*IR8a*, *21a*, *25a*, *76b*, and *93a*) and the five GluR members (*GluR1* and *2*, and *NMDAR1*, *2-1*, and *2-2*) were successfully extended by employing RNAseq and genome reads to full-length genes (sequences available in File S1). Full-length status was confirmed by comparison with orthologs from other insects (*e.g.*, Benton *et al.* 2009; Croset *et al.* 2010; Terrapon *et al.* 2014; Ioannidis *et al.* 2017), the crustaceans *Daphnia pulex* (Croset *et al.* 2010) and *Hyalella azteca* (H. M. Robertson, unpublished data), and a tick and mite (Gulia-Nuss *et al.* 2016; Hoy *et al.* 2016), as well as in BLASTP searches of the nonredundant protein database at NCBI. Some of the problems with these gene models are the result of typical difficulties with draft genome sequences, including exons in separate scaffolds or misassembled contigs, and exons partially or completely missing in intrascaffold sequence gaps in the assembly. However, in many instances, the difficulties result from the presence of noncanonical 5′ intron donor splice sites, specifically GA and GG donor sites. Spliced RNAseq reads are not shown for these introns in the Apollo genome browser at the i5k Workspace@NAL where this genome is displayed (https://i5k.nal.usda.gov/), because their mapping algorithm would not accept such unusual noncanonical splice donors, and the genes were only partially modeled by the MAKER pipeline that the authors employed for similar reasons, in addition to misassembled and missing exons. These are all long genes (19–123 kb) with large numbers of mostly short exons (19–47), providing a reasonably large sample of 297 introns (Table 1). Of these 297 introns, including an alternative-splicing arrangement in *IR25a*, 31 have GA donors while five have GG donors (12%). The sequences of these 36 noncanonical donors are provided in File S1, and sequence logos of them and the canonical GT and rare GC donors are shown in Figure 1, along with corresponding logos for the 3′ splice acceptor sites of these introns.

**Table 1:** Features of 10 conserved IR and related ionotropic GluR genes and proteins in *E. affinis*

Gene	Scaffold#	Length (bp)	Exons	Models	Amino Acids	GA/GG Donors
IR8aF	68	33,279 (26,505)	33 (23)	3	874 (569)	5/0
IR21a	3	33,150 (16,252)	23 (13)	2	763 (493)	4/1
IR25aF	269	90,005 (90,005)	34 (34)	5	907 (907)	4/2
IR76bF	532	18,715 (27,613)	19 (15)	2	480 (417)	1/0
IR93a	35	18,850 (15,580)	20 (19)	1	942 (935)	0/1
GluR1	77	69,907 (30,397)	25 (13)	2	960 (449)	3/0
GluR2	5	24,096 (14,472)	23 (14)	2	923 (615)	2/0
NMDAR1F	43	63,037 (31,051)	36 (20)	4	1095 (736)	2/0
NMDAR2-1	101	123,322 (102,094)	37 (28)	8	1055 (847)	6/1
NMDAR2-2F	141	86,450 (76,362)	47 (38)	4	1030 (823)	4/0

Lengths are from start to stop codon in large scaffolds, excluding exons present on short separate scaffolds or those that were built *de novo*, both of which presumably belong in sequence gaps in the large scaffolds, the lengths of which are included in these counts. Only coding exons are included (IR76b and NMDAR2-2 have single noncoding 5′ exons). Models are the number of models in the automated gene set available at the i5k Workspace@NAL genome browser (EAFF_v0.5.3), and usually not all exons are modeled. Numbers in parentheses are for the proteins reported in Eyun *et al.* (2017). Suffix “F” after gene name indicates that the genome assembly had to be repaired for a complete gene model to be built (details of each gene model are provided in File S1).

These GA and GG donors differ significantly from canonical donors in having AG as obligate nucleotides at the end of the preceding exon, regardless of the phase of the intron with respect to the reading frame, which is the consensus sequence for canonical 5′ donor splice sites, but is not obligate for them (Figure 1A). Almost all have A in the third position of the intron, which again is the consensus for canonical sites, but not nearly obligate. The 3′ acceptor sites do not differ as greatly between introns with these noncanonical donors *vs.* canonical donors (Figure 1B). With 12% of introns in these 10 genes having these noncanonical donor sites, the automated modeling of these long genes was compromised, and most are partial models with many exons not modeled at all (Table 1).

Once this pattern of absence of spliced RNAseq reads in the genome browser and likely GA or GG donor sites was recognized, many of these introns could be seen in a wide variety of large genes with deep RNAseq coverage in the genome browser. Full-length models were built in the Apollo browser for 26 such large genes containing GA and GG donors and encoding a wide variety of proteins (Table S1), indicating that this phenomenon is likely to be genome-wide. These 26 genes, while not a random sample, contain 623 introns interrupting their coding sequences, of which 54 have GA donors and 10 have GG donors (10% noncanonical donors), frequencies comparable with those of the 10 focus genes above. GA and GG donors have not been reported at anything like 10–12% of introns in any other eukaryote genome. Their recognition will greatly improve gene modeling in this copepod genome, but would require acceptance of only G, instead of GY, as the obligate 5′ intron donor splice site nucleotide.

The i5k pilot consortium (https://www.hgsc.bcm.edu/arthropods/i5k) has also sequenced the genome of another copepod, *Tigriopus californicus* (available in the i5k Workspace@NAL genome browser), and a genome sequence for the related *T. kingsejongensis* has been published (Kang *et al.* 2017). Neither genome evidences any GA or GG 5′ donor splice sites in homologs of these 10 receptor genes; indeed, these genes are generally far shorter with far fewer introns in these copepods, so this high frequency of GA and GG donors is restricted for now to this *Eurytemora* copepod. Alignment of the positions and phases of these GA and GG introns for the five ionotropic receptors with those of homologous genes in *T. californicus*, as well as two other available crustaceans, *H. azteca* (also available from the i5k pilot consortium) and *D. pulex* (Croset *et al.* 2010), as well as various insects (*e.g.*, Benton *et al.* 2009; Terrapon *et al.* 2014; Ioannidis *et al.* 2017) and other arthropods like a tick and mite (Gulia-Nuss *et al.* 2016; Hoy *et al.* 2016), reveals that they are unique introns in *Eurytemora*, along with many additional idiosyncratic introns with canonical donors. For example, of the 33 introns in IR25a in *E. affinis*, nine are shared with other arthropods and the remaining 24, including the six GA or GG donors, are idiosyncratic to it. It appears that this copepod underwent an explosion of intron gains, including those with noncanonical donors.

Alternative splicing of the GA donor sites in the vertebrate fibroblast growth factor receptor 1–3 genes is a complicated process involving a nearby consensus sequence (Brackenridge *et al.* 2003), but no such sequence was noticed in these copepod GA or GG donor introns, which are also not alternatively spliced but rather required for their genes to encode full-length proteins. The high frequency of these noncanonical donors implies the evolution of modified 5′ intron donor site recognition in this copepod. The only intact U1 snRNA in the genome assembly has the same highly conserved 5′ end with sequence complementing the 5′ intron donor splice consensus of AG/GTAAGT common to animals; however, recognition of the 5′ donor site is affected by other components of the snRNPs, so it is unclear how these noncanonical donor sites are recognized.

## Supplementary Material

Supplemental material is available online at www.g3journal.org/lookup/suppl/doi:10.1534/g3.117.300189/-/DC1.
