# Supplementary material for: Noncanonical GA and GG 5′ Intron Donor Splice Sites Are Common in the Copepod Eurytemora affinis
Source: G3 (Bethesda). 2017 Oct 27;7(12):3967–9. doi: 10.1534/g3.117.300189 (PMC5714493; doi:10.1534/g3.117.300189)
Supplement: Supplementary file 1 [file 3967FigureS1.docx]

**Figure S1. Sequence logos showing nucleotide frequency for the 36 non-canonical GA and GG 5’ intron donor splice sites and the 3’ acceptor sites for these introns, compared with sites for 261 introns with canonical donors in ten conserved large ionotropic glutamate receptor family genes in the copepod *Eurytemora affinis.*** A. 10 bases of exon and 13 bases of intron sequence are shown for the donors. B. 16 bases of intron and 7 bases of exon sequence are shown for the acceptors. Note the higher AT richness of the intron versus exon sequences.
