# Supplementary material for: Noncanonical GA and GG 5′ Intron Donor Splice Sites Are Common in the Copepod Eurytemora affinis
Source: G3 (Bethesda). 2017 Oct 27;7(12):3967–9. doi: 10.1534/g3.117.300189 (PMC5714493; doi:10.1534/g3.117.300189)
Supplement: Supplementary file 2 [file 3967TableS1.docx]

**Table S1. Features of 26 additional large genes containing GA and/or GG intron donors in the copepod *Eurytemora affinis*.**

**Scaffold# Length Exons Models Amino GA/GG Protein**

**(bp) acids donors**

266 9,353 16 1 412 1/0 Prolyl 4-hydroxylase subunit alpha1

266 47,184 44 4 1389 5/0 Cytoplasmic alanine-tRNA ligase

340 20,651 25 2 638 2/1 Matrix metalloproteinase

340 34,537 10 1 417 1/0 Unknown protein

150 35,272 48 3 1,942 1/0 Vitellogenin receptor

150 25,303 22 3 619 1/0 Unknown protein

150 53,057 25 3 827 4/1 PHD_SF superfamily

502 46,049 28 4 1,098 2/1 Dentin sialophosphoprotein*

76 22,714 19 3 908 2/0 Na/K transporting ATPase subunit alpha*

76 7,658 12 2 862 1/0 Neur_chan_LBD superfamily

535 30,352 34 3 1,951 1/0 Med. of RNA pol. II transcript. subunit 13

535 28,359 33 1 1,076 1/1 Prophenoloxidase

535 29,003 27 2 1,231 3/0 MDNI superfamily

535 41,780 31 2 752 2/0 Cystolic carboxypeptidase 6

499 6,367 7 1 148 1/0 Microsomal glutathione S-transferase

499 24,414 26 2 728 2/0 Probable zinc finger protein

156 10,293 13 1 313 1/0 3’(2’),5’-bisphosphate nucleotidase 1

156 34,889 22 1 623 0/1 DUF1446 superfamily

156 13,681 19 2 591 2/0 F-box domain protein

156 15,495 22 1 594 2/0 Endoglycoceramidase

273 30,253 29 3 812 6/1 Neurochondrin

273 33,499 38 4 1,463 5/0 Unknown protein

243 17,477 24 3 702 2/1 HEAT_2 superfamily

307 36,431 35 5 1,084 3/3 Nuclear pore complex protein Nup133

501 9,558 14 1 613 1/0 ATP-dependent RNA helicase pitchoune

501 7,652 26 2 2,544 2/0 Small subunit processome component 20

These genes were noticed as neighbors of members of the arthropod gustatory receptor family in various scaffolds. My focus was on large genes with deep RNAseq coverage and apparent GA and/or GG donors to show the diversity of genes with these non-canonical donors, but there are many shorter genes, like those encoding gustatory receptors, without such donors. Likely GA or GG donors were confirmed by searching the SRA for spliced RNAseq reads. All of these models were built and annotated in the i5k Workspace@NAL Apollo genome browser. Gene length includes 5’ and 3’ UTR regions, which commonly include additional non-coding exons in these genes. The number of exons concerns only coding exons. Models are the number of models in the automated gene set available at the i5k Workspace@NAL genome browser (EAFF_v0.5.3), and usually not all exons are modeled (all single models were incomplete in some way). “Unknown proteins” had no significant matches in the non-redundant protein database at NCBI in BLASTP searches with default settings. When a protein had matches only to a superfamily those are indicated. An asterisk after the protein name indicates that the N-terminus is incomplete.
