# Supplementary material for: Noncanonical GA and GG 5′ Intron Donor Splice Sites Are Common in the Copepod Eurytemora affinis
Source: G3 (Bethesda). 2017 Oct 27;7(12):3967–9. doi: 10.1534/g3.117.300189 (PMC5714493; doi:10.1534/g3.117.300189)
Supplement: Supplementary file 3 [file 3967FileS1.docx]

**Supplementary File 1**

**Notes on each gene** (suffix F indicates genes requiring repair of the assembly)

**IR8aF** – Eyun et al. have their first 45 amino acids encoded by three unsupported exons within real introns, and miss the first half of this protein. The genome assembly has a gap interrupting exon 10.

**IR21a** – Eyun et al. have an unalignable first short exon encoding 6 amino acids, skip exon 16, and end with 88 amino acids encoded by an intron, they therefore miss both the front and back of this protein.

**IR25aF** – Eyun et al. have the protein sequence correct, but like other genes derived it from a transcript as the genome assembly has multiple problems. Specifically, exons 5, 26, 29, and 31 are missing in gaps in the assembly, exon 21 is in a separate short scaffold, and exon 9 is on a misassembled contig upstream of exons 7 and 8. IR25a is also unusual in having an alternatively-spliced version near the C-terminus, which has an alternative for exon 29 split into three exons (one of these two novel introns has a GA donor) and encodes somewhat different amino acids including two extra ones.

**IR76bF** – Eyun et al. have three upstream exons, and an exon 5 that have no RNAseq support and no similarity to other IR76b proteins, so their origins are enigmatic. They also skip the short exon 5 with a GA donor, include flanking intronic sequence in exon 12, skip exon 13 (which is not in the genome assembly), and then skip exons 14-19 to a far downstream exon that does not belong in this gene.

**IR93a** – Eyun et al. miss a short upstream coding exon.

**GluR1** – Eyun et al. begin their protein in exon 13, hence it is only the C-terminal half.

**GluR2** – Eyun et al. end their protein at an alternatively-spliced cassette exon, missing the last nine exons encoding the C-terminus.

**NMDAR1F** – Eyun et al. begin their protein in exon 16, hence miss the N-terminal third of this protein. The genome assembly has exon 4 in a short separate scaffold, exons 5, 8, 9, and 15 are missing from the assembly, and exon 18 is misassembled in front of exon 17.

**NMDAR2-1** – Eyun et al. end their protein within intron 28, missing the C-terminal quarter of the protein.

**NMDAR2-2F** – Eyun et al. end their protein within exon 38, missing the C-terminal quarter of the protein. The genome assembly is missing the end of exon 7 and the start of exon 8 in a gap, exons 36, 37, and 43 are missing, and exons 38, 39, and 40 are misassembled within 681kb scaffold246, with exon 39 misassembled within that scaffold in front of exon 38.

**Full-length sequences of five ionotropic receptors and five ionotropic glutamate receptors from the copepod *Eurytemora affinis*.** Suffix F indicates the genome assembly had to be repaired to build the complete gene model.

>IR8aF

MLFLLIFSTVFSVGYCQTSIITVVEKDELAVLKSDIAKNIATVEVSTSQTIVLKTIEFTRSESMALNKTMCNQLTAEPYSAVLDLTWGGWIRGRQNADMIGLPYYRVDITNHPYVMAVAALFNQSQVVDSVFVFESKQELDMCMYYIIMYSELRITGAWLAGDKASLNKLRDMKPRPSNIVVIGSKTAVSDILAALDVRELIKRDSFYSLIYTDLSTPASVTEDISQYFQSNMSVRLFTPQQSTCCQLIGSSEPDCVCDKTIPRLFLNAALEVVGKATKVLNSVQTINQKWVCGSNTSIPTRTKLNTEIATQAKERGLYFGLDATNVPLLSPSFKLDVTLFTPQSKQLLTIGNWYMGGGYNTSTTYKEVVMKRSFRIGVVLGKPWAWVSGAKEDKQTNPKSINNALKNKGLEGYTLDLLDEVSKAMHFDYEIVIATGNDYGARDPVTSQWSGLMGDLISGNIDISLASLIMTTEREEYVDFVTPYFDQSGISILLREKYVERNMFKFFLVLQDEVWLGILGCVLVVILILWILDRFSPYSYVNNREAYPEGAREFTFGECIWFCLTSLTPQGGGECPKALSARVLVAAYWLFIVLMLATFTSNLAALLTVERMQTTVQSLEDLARQSQVNYTVLEGSPTMEYFKNMAGAEEELYRKWKELTLNSSAESNKFRVWDYPIREQYTHIWDVIKSGNPVKSPEEGYNRVKESQKGLFAFISDASEIKYQYYTNCEFLEIGEPFAEQPLAIAVQEGSSLSKELSQVVLGLQKDRFFEKVHSKFWNIGQRQACPVMNDNQGITLESLGGIFLTTLVGLALSVLMLIYEIYQEKKEEQIAKIQPMLFKNRQDEKMYGDLDKPTYTLSGDMTRSSRKLADVD

>IR21a

MEFFPGRKSRIKFYPRRKSRTELVLILVSFLLLVPQSHATKGHGIGDVLNNIVQFYNTDVHFISDFSDGDMYDVAPKISEAGKPIFMYNIDDLVARHLALPLEKCPVFGGAHGDTGHGDDNGDDHGDHNEDDHGDDHGDDHGDDYGDEHNNHEDDGHYKEKNMAENLTTIAYMFPNSFVEPSVQYSEKLREDEDIPKSVLYRRPSLLSRGVYHKTIVVWVQDNQRVLNLFRNTALFCNQKIDVGIYNMDDRFIFIVSSDTVRDQILLDPYITKHSSVAVIRPLTGLEDKFIVFTYNFFHAANNKPQILTRFLWSPVNMFKNKQQIFTPVNTFDGHKFKVSTLPWSHHMLGDQVEAPEGTPATYTNYWGYEFDTLKFLSKHLDFTYEIFNPQDGKWGHIEADGKYSGMIGKVALKDYDFIISDIFITYIRVQILDGTVAFDKDYMVFVAPNPQKEPKYLAIIKPLDPFVWLLLVCSWGATTIVFVIIARIEEKVMDTEIRIFNKFYDALYMVYGCIIGDGFNKNINYNTLKANAVRCLLAFWGLYCFIINSAYGGNLKAFLTNPGTGQPIDTLSDVLKSGLPWGMVLYGEEEEEMMASSPDPVIRRIWDDKDVKPYSPTPQIGPVRDGQGIFIDWKSGLEPAIYSKYSTPGGDPLVHISSTPVFMPNFAGWGFHKFNPWKVTFDEAIQRLIEAGLINEYKKRTWVRMKQEAVLAGEVDEYVIPPAIAPIYMEDMQSAFYLLSLFSLSAFVVFFTELLIKKYKII

>IR25aF

MVLQLLAGLPSILLLITPTFALNVYVVSDKNNGIADDSTKVAFDYLASRPTDPVTINAKKFSTLEGTDPKVVIDKVCGDLDAMIDGGQTPDLLLDLTRGGVNSEVVKSLSLTLGIPTVTATYGSVGDIREWKELTMEQSKYLIQVRPPGDTLPDIIRSLVTDLNITNAGILYDESFVMDHKYRSLLLNLPTRHIIKPLETTDAGIKDQLTRLESVDINNFFVLASLANLKMVYGVASRKGMTGEKYAWFAGTKDSGQEFDQTCCERMKVAFFHASPIPSNIMRSLKTMKNGLTGMPEVDAGFYFDLAVRAATSMKKLMAGGSLPNYSLSKCSEYTETTPVARSIDLLSVFKVDNPGMSYAYGPMKISSNGLSMMNFKVNLLSYTIESMKVADRKEIGSWEWGSSIMYSPGMMNSMSGFRATTVYRVVTVLQAPFMMMDEQGEYSGYCVDLINELKDMMGFQYEIKVVEDYGNMDMDMSWNGMIRELVDKRADIALGALSVMAERENVVDFTVPYYDLVGISILMKQPQVPTSLFKFLSVLEDSVWFCILAAYVVTSVIMWIFDRWSPYSYQNNMEKYIDDDEKRYFNLKECLWFCMTSLTPQGGGEAPKNLSGRLVAATWWLFGFIIIASYTANLAAFLTVSRLETPVESLEDLAKQYKIKYAPKNGSSAAVYFQRMAYIEERFYEIWKDMSLNDSLNELERSKLAVWDYPVSDKYTKIWQSMQEAKLPDTDEEAYARVRASPSSSEGFAFLGDATDIRYQTLTNCDLQMVGEEFSRKPYALAVQQGSPLKDQLNDAILKLLNQRKLETLKERWWNQNPKKKVCDANEEDGGGISIHNIGGVFIVIFVGIGLAIITLGIEYWYYKYKKPLSRVSSSAQNIIDKKGHAFDKSYGGRGSADVVPVGNPW

>IR76bF

MSILNYWKSLSNEKEKSDFMVTKHITVGSVVDVDPIHLTEKKNGMRIYKFGVLKEIFDAFSSTYNMSYNIIPTLDDSYGVDVGNGSFSGVIGMVQRKEVDVGGGIFSRMEERQKVVDFSAAIVEAMNIMWVNVDLADTDYIIAPFTNIVWLYVLIAAFLMGPIMYIMINCFERNVLLDTPPQYIGLMNYYWFLYGALVKQGSSIVPSTDVMRILFLTWWIYITFLTAEYTAQLTANLTKNNRTLPIHHLQDMSSDPSFKWATPGNGSFQTMIDSWPDFVNLKNDIISGKGRYIGSAATDGIKFLEGGPSRFLVAPTYEIDSIVFEDYKKEGKREGSSACTYSQLNKYTFGKVSLGLIFPKESRQLQEMFNKVLQKLLQSGLIEHSTDVTSPNIDLESACKEVVGMEPKKIFLYEVSMTFQMMLGGGIVAAIVFGLEILCLPLWEEIKLQMKGCKRFMREEKRRRRRLNKIHVHLVQHKI

>IR93a

MSQRMKAMLLLLFLCVLSTRCVVDAQSETSTADLVPELELALDPPDETMEDELMDDGSMDDGSMDNGTQSVIIDPGLSWETLASLPNFTHIKLGVVINQRWIGGPMNVMKTIIKDNIEQAEANVLKNGKIDIFWSDEDEINDDYGNNLTAVLSFVNCKESHSLSSVLESKPVLYLAITDIGCKRFEVGDQLMFPLVEYARGIVQLLADLRYSNTIQWDSYIVIHDESLDKELEGEVYNVLANGKSGEEEMLMSSSQHRNDVAVSMFDLGTINNMSASDRNMKLGNLLKGFPGKDLGNHFLIICKHSIVQDMMNIAKSNKMFNFDSQWVFMVTDTNSSSFDMSPYIKMASDGYNLGFVFNTSIALPGIVCPTGIECVIKSTIDNMIQSFQNILENEFKIFDIVTIEEWDIIKPSPEERSMAIVEDIKLRIEETKGQCNNCTKWMMEAVEVREANRINQLEVGTWNPTLGLSTVDDLLPHVTGGFRGRAITVGSLHYPPWTIFERNDRGQITKYSGLIFSLLNEISYKLNFTYVVKEPSDGKWGDLKNGKWNGMISQVINNEVLLAAAAFSVSAEREVVVNFTEPIDMQPYAFMYRRPKELSRYLLFIDPFTPSVWAYIAAMTAIIGPIFWVIHRYSYFYKYYDAVKPFGLFRVEECVWFVYAALIQQGGTILPTADSGRILIAFWWLFVIVVVTTYSGNLVAFLTFPQIEFPLNTLDDLIKKGETDGITWGLLQDSVIEAYFRDAEEPKFKFIGDHAMRHTEPGTDPNSEVFQLIKNEEHVYIEWKSKLEVMMKEQYNITRECDYALGKENFFFERVALAFPEDSPWLHKFNKVIRDVLQAGLNQKWKQDFWPSDDECSTTASGGTGTTAVVTVMDMQGSFYILFFGCLFGLIFLGIEHLIKNKMDAKGKSPIQPFTP

>GluR1

MRNRLCSTMSNKLLILLVFFQTPVSYSKLRIPIGTIFSKAPDWQDVFDAMNLAMEGHSSANSSTEFDMKFYVDNIDTVDAYKLTKIICKQFQRGAFSLMGSVDPESFDTLHSFSNAFEMPFVTPWFPEKKSDSSDGSLDFAVQMQPDYHQTIVDLIVHYNWNSVIYIYHSLEGLHRLQKIYKSIPKNGFGSPVFYVQTAKKIETAEEGVEFLKELEYLDRESIKHIILDCPANIAKQMIVKHVQTIVLGRRNYHYLMSGLVLDEVWEGSVQEYGAINITGLRIVQYGSDISKHFFSRWKSLEKIPRGRKNSISAEGALAFDIVNLLISSFNNLLRQTPDAFKPKKKSRVECGDMSGQEKPGTWEHGQTILKFIRSAQLQGLTGMISFDRQGRRYNFSLDVLEMTSRSELVQIGTWSDTIGLSLASSPIIRNHPVQTKKLTMISFDRQGRRYNFSLDVLEMTSRSELVQIGTWSDTIGLSLASSPIIRNHPVQTKKLTSQTFVMTTILEEPYMMLAKPRKGETLVGNDRYEGYCKDLATLITSLTGIKFQIKPVNDSKYGSPDPLAPGGWNGMVGELVRREAEIAIAPLTINSQREQVADFTKPFMSLGISIMIKEPVKQRPGVFTFMNPLSLEIWMCVLFAYIGVSIVLFLVSRFSPYEWQIEESIAGTTVSNHFSVINSLWFALGAFMQQGIDITPRSVSGRIVGGVWWFFSLILISSYTANLAAFLTVDRMVAPINSADDLAAQTEIEYGALDGGSTVAFLKNSKISVYSRMWEFMSSRPSVLALSTAEGILRVRESKGKYAFLIESSTNEYVNERQPCDTMKVGKNLDAKGYGIATSRGSGLKDIINLAVLNLTENGDLAKLKNRWWYDRSECKKEKEKAAKSELSLSHVAGIFYILICGLVLAMITALIEFCYKASTESKKAKVPMSDAMKNKARLALSGGRDIDRIMLYGDSSAL

>GluR2 MTVNQKFPSFLQIAFLILLSSKLTSSALPPVIKIAALFDETHGKSQELAFHRAVKMVNDDRTILTRSLVSPDIGNYPWDDSFKASKKLCELIMPGVAAIFGPTSPTASNHVQSVSDALHLPFLETRWDYDFQRSPFSINVHPHPSMLGKAYADFVHIVDWKSFVILYESEEGLVRLQELIKLPKTFSDIRVTLRQLPHGTTDYRPLLKEIKKSEETKIVLDCSFDNLERILSQANELHLLTDYHSYLVTSLDVDKINLSPYTNQNVNISGFSLTASGTRAIESYLKEFPNTGRGKEHMLFSENALVYDAVWTFAKALNDLDSLQSIQLEPLSCEQPGPWADGEKVLSYLKEVDHLGLTGEIKFDADGYRTDFQLELMEKMRSRTKKTGIWTAQGGVNYTLTATEIEGQMVEKLQNKTLKITTALTEPFVIERIFDYPVSPEAKERMSFEERFEGFCVDLIKELSKEVKFKYKFQLEPSGSYGSFKNGKWTGMIAELRSQQADMAAIDMSITSIRQRAVDFTMPFMNTGVGILYKKKKPPAPNLFSFLSPLSLDVWIYMTTAYLGVSILMFLLARISPYEWNESGERELSNNFNISNALWFGIGSFLCQGCDILPKAISTRMVAGMWWFFTLIMISSYTANLAAFLTAAKMDSPINSAEDLAKQTKIKYGTYCCGSTNAFFQGSTIPTYQKINAYMESTKPSVYTTGNSQGLDRVLKEDGMYAFFMEAAAIEYHVERKCDLKQLGGLLDSKGYGIALPKDSPYTAAMSAGVLRLQESGKLQELKIKWWKNERGGGSCSGDAAGNSAQLDLASLGGVFIVLIGGMVVSIIIAIFEFTWKQRKLAVDENESVWAEMWEELKFAVNFRAGDTKPIKRESSRAASKSLLSKSKAESLNKYGVIGDEAKSIKSRGSRKDSNYACFNDNY

>NMDAR1F

MVSRTLVSLPLHLPPLPFILLLFLPTNPGSSAINKPKTLYIGGVLSTTATAQAFTMEAQHIDYQHLYLPENVSLYDSTQIMDSNPIKTAIIVCNKLIKERVYAIIVSKPENGDLSPASVSYTAGFYHIPVLGISSRDSAFSDKNIHVSFLRTVPPYSHQADVWVELLKHFHYRQIVFVHSSDTDGRSLLGRFQTRAQDTYNEDQEVKVMVEKVEEFEPGQESYSSTIANLKDCQARVYFLYATKQDAEKIFADAAEQNMTGTGYAWIVTEQALKSKNIPNGTLGLVLTRSQDEKAHIHDALHILARALAKLYEEEEDVEEAPSNCNSSGSIWKTGKKLFDKIREQKLEDGRTGRVVFDAMGDRLFAEYKVMNIQPNEAGEKELVAVGNYSFSRETTGMVLSIEDSLIIWPGGNRDKPTGVMIPTHLKVLTIVERPFIEHMNNTSGCEREDGWYPCPEYVFSSNSTLLTTMGSKYCCKGYCMDLLEKLALKCNFTYDVYLSLDGDYGSLERNNLTGKQEWTGLIGELVTERADMIVAPLTINPERAQVMEFSKPFKYQGITILQKRQPRASQLVSFLQPFKPTLWVLVLVSVKVIAVCLYLLDRFSPFGRYNTDSGEVREEDSLNLTAAIWFAWGVLLNSGIGEGTPRSFSARVLGMVWAGFAMIIVASYTANLAAFLVLDKPQTSLTGINDPRLRNPMENFTYATVKGSSVDMYFRRQVELSNMYRTMEGKHFRTPELAIDAVRNGSLKAFIWDSSRLEYEAARDCDLITAGELFGRSGYGVGLQKGSPWADQVTLAILDFHESGYMERLDNKWILSSEEDNICNEKDSNSPATLGLENMMGVFILVGAGIVGGIGLILLEIIYHKHKMRKVERSEAAKVAISRWKGTVEKRKTIRESKVERSKKQTSKTNGTSIIRDNLSLSFDNLTKGSKDSRWQRVTHLVRPRSTATGLCQSPLTPRYMRSLPSAEFLRMVENMEHHSSPIAPLSVDFNKKRRKRNPPDSPDILQRCTPPPDINEGVSVNSPSEYISSDYAPVSTDGWVPPIENLPCPPPPPRNRLKSYSGAYRGGQSSVLSPPPYRRQYSDNSYSVDSEIV

>NMDAR2-1

MFVFLMTFFSEIMIFSLFSLILLPVSGLDISSNDNFGRGKGRNTIGNINKPTLNLGLILPQSVFKEKQYRAAVIEAVDLVKNKRRPAFRFLNDYHLDDRQIHFSMMSVNPSPTVILENLCETFLENNVTAIIYMTNSEMFGRSTAASQYFLQLAGYLGIPVIAWNADNSGLQSQSRLRVQLAPSLEHQVEAMLALLIRYNWQQFSVVTSDIAGHDDFIQAVRDKVVYFRDKMNFKFFVQAEVKVNSQEDMNTLTNAETRIILLYSTKEEARHIMSWADQAGLTGTNYVWIVTQSVIGESEGGSAEALTQFPIGMLGVSFPTSFQALIDQIPVALSVYAEGVERLLSTPLNGTLMPHLSCTEGTKSTEASWDLGETFYKHIKSVRMAGSAGKPEVKFLADGSLSNVELMVMNLRKPTSGSSVQHIWEKIGVWKSWKSEAHGLEIKDIIWPGDSHVPPQGVPEKFHLTVGFLEEPPFINIAPPDPVSGKCNVDRGIRCRMENSTEAEKKNGTEQFQCCSGFCVDLLAKFAEDLQFEFDLVRVQDPKWGSIVNGKWNGLMLELVSKKFDMVLTSLKINAERESVVDFTAPFLESGTAILVAKRTGIISPTAFLEPFDAASWLLIAFAAVQISALTIFFFEWLSPAGYNMKTSSEPDHKFSICRTFWMVWALLFQAPVQMDCPRAFTARFMASVWALFAVVFLAIYTANLAAFMITREEWDQFQGIDDTRLTNPQSMKPPLRFGTVPWTYTENAIRRHFPNMHMYMKAFNKENVMEGVEAVKKGELDAFIYDGTVLEYIVGQDDECRLLTVGNWYAMTGYGVAFPRHSKHFPNFNKKVMDYSENGDLERLRRFWLTGTCRPRKQEKRSSEPLAPEQFLSAFFLLLLGVCLAAGLCSFEYLYCHYIRSKIARRDTPGCCSLISQSMGASLTMKGTVLEASAMLSRHKCPDPTCDTILWKVKRELDLTRLKLSELKAEVEARGLEKTSNPSTHQERIFEVTEDFLTETIVERCSTDLGCSTDSARISDDSSDKTSFIEQHASKRDIFRRYSQGFSEIETVL

>NMDAR2-2F

MLWCKVLFLLTLEGCGGIDIYGNRKGGRRPEHSIIYSEETFDHRVFLKLGIIVPHTQWLERKRRYLQIISSSLSNLDRNVLLAINESYRFEFSRRQELRAPDDSINFDEVVAVSPPPRDILDLLCDQIRNSSAAIIYLTDTERYGRSTASSQYFLQLAAYLGIPVIAWNPDNSGLEKRTFQDGQKSIGKLQLAPTIEHQVSAMLSILVRYNWHQFGIVTSEIAGHDDFVQTFREKIPGYQDTFRFIIQDVFKLTESKNYTVEDLAKSEVRIFLLYSTQREAELIMQKGAAAGLTGSNYLWIVTQSVVGDPKDKVSLREKFPIGMLGIHFIVDLDTVLHHIVPLAVQIFSYGAMSLVRSNRGRLNYDLSCEGNITNWDLGNEFYRHLLNVTVPSYDKEPVFKPDGTTRHVTLKIVNLRPTIEHKMKWEEVGSWGTRTGLDIKDIVWPDNSHVPPQGVPEKFSMKITFLEEPPFIIVSEPDPVSGRCSMNRGVACLTRSVENEKSENETLTRMCCSGLLIDLLRKFEDDLGFNYDLIRVDDPKWGTLENGKWNGLMGVLVNKKTDMVLSSLKISREREKDVDFTVPFLESGIAIVVAKRTGIISPTAFLEPFDINSWMLVVLVAVQAAALSIFIFEWLSPAGYDMKVLPTKTGHRFSLFRTYWLVCAVLFQASVQVDCPRGLTSRFMSSIWALFAVVFLAIYTANLAAFMIPRKEYHDLTGLTDSRISNPQSHKPPLKFTTLPYSHAYVTLQKYHAEVYDHIKSNKLIFNTSEHAVQAVKEGLLDAFIYDGTVLSYLVSQDEECRLLQVGSWSAMTGYGLAFPTNSKYRTMFNDKLLDYRENGDLERLGRFWLHGTCKPNEQEKRASEPLSEAQFLSAFLLLVCGIITSIFLLTCEHIYLRYFSPTLREKTIYNSWVSIFNKDGASQSRNQLVRNCSIPEEIAGEWENKQREGSLASTKLCSTRRTSVRSFLDLGYGDQNCGEDVCKTKLRRLRGDLDCARDQIRMLEDQLNKHGIPLRRTSKEIAEKETVL

**Sequences of the 36 GA and GG 5’ splice site donors**. Preceding exons are in capitals and intron sequences are in lower case. Sequences of the 261 canonical donors and all acceptor sites used to create the sequence logos in Figure 1 are available on request from the author.

IR8a_Intron8 ATCTATACAGgaatactatttga

IR8a_Intron16 ATATCTCTAGgaaaaaatacaaa

IR8a_Intron19 TTAACAACAGgaaacacatttga

IR8a_Intron21 CTACTTACAGgaaaccattgtta

IR8a_Intron24 ACCCAATCAGgaaaataaaaata

IR21a_Intron2 CAGATTTCAGgaaattaaatttt

IR21a_Intron4 ACACTACAAGgacaaaattaaca

IR21a_Intron6 TATACCAAAGgaatttcaaataa

IR21a_Intron10 TACTCACCAGgaaggatgctcaa

IR21a_Intron22 GGACATGCAGggagacacagtat

IR25a_Intron4 AATCTCTTAGggaggtcgcagtc

IR25a_Intron8 TCCCAACTAGgaacaatattaaa

IR25a_Intron9 AACTAACCAGggattaaggatta

IR25a_Intron12 CTTATGGCAGgaatcggatctaa

IR25a_Intron18 GAGCACTCAGgaatataatgatt

IR25a_IntronALT TCCTATCAAGgaattgtattact

IR76b_Intron5 GCTGCAATAGgaacaaatcaata

IR93a_Intron2 ATTGATCCAGggaaccagtttaa

GluR1_Intron3 CTACCATCAGgaaaccattttat

GluR1_Intron8 CCGGACTCAGgaatttagatatt

GluR1_Intron22 TTCGTGTTAGgaatttaaatcaa

GluR2_Intron4 CTCCTTTTAGgaacattacttta

GluR2_Intron7 GAATACTTAGgaatgtcgtcttc

NMDAR1_Intron3 TTCTACACAGgaaaaataaaaac

NMDAR1_Intron19 ACAACACTAGgaacgtattctat

NMDAR2-1_Intron1 TTGACATTAGgaaagcaaacaat

NMDAR2-1_Intron10 CAGTACAAAGgaacacagatatc

NMDAR2-1_Intron11 AGTGTCATAGgaaatcatttcaa

NMDAR2-1_Intron18 TCTTGCCAAGgaaaacaacgaat

NMDAR2-1_Intron25 ATGCTATCAGggaacattaattc

NMDAR2-1_Intron30 TTAGCTGCAGgaaaacaacttta

NMDAR2-1_Intron34 AACTCATCAGgaatatactagtt

NMDAR2-2_Intron2 CGTCGACCAGgaaaagaaaaaag

NMDAR2-2_Intron22 TCCACCTCAGgaatataatctac

NMDAR2-2_Intron26 ATCTTATCAGgaacgcatataac

NMDAR2-2_Intron40 TTTACCTCAGgaatattcacttt
